# Reimagining natural hazards and disaster preparedness: charting a new course for the future

**DOI:** 10.1186/s12889-023-15497-y

**Published:** 2023-03-28

**Authors:** Krzysztof Goniewicz, Md Nazirul Islam Sarker, Monica Schoch-Spana

**Affiliations:** 1 Department of Security Studies, Polish Air Force University, Deblin, Poland; 2 School of Social Sciences, Universiti Sains Malaysia, Pulau Pinang, Malaysia; 3 Department of Development Studies, Daffodil International University, Dhaka, Bangladesh; 4 Johns Hopkins University, Baltimore, USA

The world is facing unprecedented challenges from disasters and natural hazards, which are increasing in frequency and intensity due to climate change. Moreover, they can overlap, producing compounded and cascading effects [1, 2]. While these events alone and together can be devastating, the good news is that we can take steps to mitigate their impact and prepare for their aftermath. This is where the art of disaster preparedness comes into play.

In this collection, we explore the latest research and best practices for preparing for natural hazards and disasters. We welcome submissions that showcase innovative solutions and strategies for dealing with a range of hazards, including hurricanes, earthquakes, floods, and wildfires.

One of the key messages from the collection is the importance of preparedness; anticipation and preemption are powerful interventions. Disaster preparedness is crucial for health security for several reasons. Disasters and emergencies can place an enormous strain on health infrastructure, including hospitals, clinics, and medical supply chains [3]. By being prepared, communities can take steps to protect this infrastructure and ensure that it is able to function during and after a disaster. Disaster preparedness can help to ensure that emergency responders are able to provide a timely response to emergencies. This can be critical in situations where every minute counts, such as during a disaster or a terrorist attack [4–6].

Another point is prevention of disease outbreaks. Disasters can increase the risk of disease outbreaks, particularly in areas where sanitation and hygiene are compromised. Preparedness efforts can help to reduce the risk of outbreaks by ensuring that individuals have access to clean water, adequate sanitation facilities, and proper medical care [7]. At the same time, readiness for infectious disease outbreaks that can complicate established response measures such as sheltering and evacuation is essential [8].

Disasters can also have a significant impact on mental health, causing stress, anxiety, and depression [9, 10]. Preparedness efforts can include providing support for mental health needs, which can help individuals to cope with the effects of a disaster [11, 12]. Overall, disaster preparedness is a critical component of health security. By being prepared, communities can help to reduce the impact of disasters on health and wellbeing, and ensure that individuals have access to the medical care and support they need during and after a disaster.

Another theme that we would like to see emerging from the collection of articles is the need for collaboration and cooperation [13–15]. In order to prepare for and respond to disasters effectively, individuals, organizations, and governments must work together. This can involve sharing resources and expertise, coordinating efforts, and engaging in community outreach to ensure that everyone is on the same page.

Training for mass casualty incidents and disasters should be conducted regularly and refreshed at intervals. In order to improve society preparedness and resiliency, disaster management competencies should be linked to the overall quality improvement process [16, 17].

In order to ensure the appropriate level of knowledge and skills of society for mass casualty incidents and disasters, it is necessary (as a minimum) to design training curriculum that is comprehensive and aligns with international standards that include the roles, responsibilities, functions and resources needed for MCI and disaster preparedness and response [18, 19]. The provision of training for mass casualty incidents and disasters by the employer should be mandatory, as should the participation of employees [20–23].

This collection aims to provide an up-to-date overview of the latest scientific research and innovative solutions related to preparing for and responding to natural hazards. The collection is highly relevant to researchers, policy makers, and other experts in the field of disaster management, as they offer insights into emerging trends, challenges, and best practices for addressing natural hazards. We are interested in articles that provide practical advice and strategies that can be implemented in real-world situations, such as community outreach programs, emergency response plans, and risk reduction measures. By implementing these strategies, communities can build resilience and better navigate the challenges posed by natural hazards.

We believe this collection is an essential resource for anyone involved in disaster research and management. It aims to provide a comprehensive overview of the latest research and best practices for preparing for and responding to natural hazards. We are interested in submissions that contribute to this goal by presenting practical solutions and strategies that can be implemented in a range of settings. By working together and implementing the strategies outlined in this collection, we can build more resilient communities and navigate natural hazards with confidence based on the latest science.

## Data Availability

Not applicable.
